# Perioperative blood transfusion and adverse events in total hip and knee arthroplasty: a TriNetX-based analysis

**DOI:** 10.3389/fmed.2026.1583518

**Published:** 2026-01-22

**Authors:** Hsien-Hsu Hsieh, Jun-Hong Lin, Huey-En Tzeng, Ju-Huei Chien, Tsing-Fen Ho

**Affiliations:** 1 Division of General Laboratory, Department of Pathology and Laboratory Medicine, Taichung Veterans General Hospital, Taichung, Taiwan; 2 Department of Medical Laboratory Science and Biotechnology, Central Taiwan University of Science and Technology, Taichung, Taiwan; 3 Division of Transfusion Medicine, Department of Pathology and Laboratory Medicine, Taichung Veterans General Hospital, Taichung, Taiwan; 4 Chief of Division of Medical Oncology, Department of Oncology, Taichung Veterans General Hospital, Taichung, Taiwan; 5 Department of Post-Baccalaureate Medicine, College of Medicine, National Chung Hsing University, Taichung, Taiwan; 6 Department of Research, Taichung Tzu-Chi Hospital, Buddhist Tzu-Chi Medical Foundation, Taichung, Taiwan

**Keywords:** allogeneic blood transfusion, deep vein thrombosis, patient blood management, perioperative complications, postoperative infections, pulmonary embolism, total hip arthroplasty, total knee arthroplasty

## Abstract

**Background:**

Life expectancy has increased globally, leading to a higher prevalence of age-related and metabolic diseases and, consequently, a rising incidence of osteoarthritis among older adults. Total knee arthroplasty (TKA) and total hip arthroplasty (THA) are well-established surgical treatments for advanced osteoarthritis. Despite advances in perioperative management, blood loss remains a major concern and often necessitates allogeneic blood transfusion. This study used the TriNetX global research database to investigate the association between perioperative allogeneic blood transfusion and adverse postoperative outcomes.

**Methods:**

This retrospective cohort study analyzed de-identified data from the TriNetX network, a global clinical research platform. Patients who underwent TKA or THA between January 2019 and September 2024 were included. Exclusion criteria were age younger than 18 years and documented infections within 3 days prior to surgery. A total of 323,669 cases were identified. Patients were categorized into perioperative transfusion and non-transfusion groups, and propensity score matching was performed at a 1:1 ratio, yielding 2,040 matched pairs. Primary outcomes included postoperative transfusion, infection-related complications, thromboembolic events, and 30-day mortality. Statistical analyses were conducted using chi-square tests, *t*-tests, and Cox proportional-hazards models to estimate hazard ratios (HRs) and 95% confidence intervals (CIs).

**Results:**

The incidence of perioperative allogeneic blood transfusion was 0.63% (*n* = 2,041). Perioperative blood transfusion was associated with a significantly increased risk of adverse outcomes within 30 days after total joint replacement, including postoperative transfusion (HR = 9.67, *p* < 0.001), procedural infections (HR = 3.82, *p* < 0.001), sepsis (HR = 2.30, *p* = 0.001), deep vein thrombosis (HR = 2.20, *p* = 0.002), and 30-day mortality (HR = 2.90, *p* = 0.002).

**Conclusion:**

Perioperative allogeneic blood transfusion in patients undergoing TKA or THA is associated with an increased risk of postoperative transfusion, infectious complications, thromboembolic events, and mortality. These findings underscore the importance of implementing patient blood management and blood conservation strategies to mitigate transfusion-related complications.

## 1 Introduction

The global population is aging rapidly, and in the aftermath of the COVID-19 pandemic, global life expectancy has resumed an upward trajectory. In 2024, life expectancy at birth reached 73.3 years, reflecting an increase of 8.4 years compared with that in 1995. By 2054, the global average life expectancy is expected to further increase to 77.4 years (1). This demographic shift is reshaping health-care systems worldwide, presenting both challenges and opportunities for transformation.

Degenerative arthritis is the most prevalent joint disease among older adults. In its early stages, this condition can be managed through lifestyle modifications, pharmacological treatments, and physical therapy. However, as the disease progresses and joint pain becomes severe and refractory to conservative measures, total joint replacement surgery becomes a necessary intervention to improve patient quality of life. Joint replacement procedures have become increasingly common in aging populations, and total knee arthroplasty (TKA) and total hip arthroplasty (THA) are the most frequently performed procedures. TKA is primarily indicated for degenerative osteoarthritis, rheumatoid arthritis (2), and post-traumatic arthritis (3), whereas THA is commonly performed for hip osteoarthritis and femoral neck fractures (4). Projections based on regression models and US Census Bureau data indicate that by 2030, the annual demand for primary THA will increase by 174%, reaching 572,000 cases, whereas the demand for primary TKA will increase by 673%, reaching 3.48 million cases. In addition, the demand for revision surgery is expected to rise substantially, with revision THA projected to double by 2026, reaching 96,700 cases annually (5).

Both THA and TKA are associated with substantial perioperative blood loss and may necessitate blood transfusion. Previous studies have reported that patients who undergo blood transfusions experience higher rates of postoperative complications than those who do not (6). Furthermore, allogeneic blood transfusion may affect immune function through transfusion-related immunomodulation (TRIM), potentially increasing the risk of acute infections (7, 8). Primary THA and TKA are among the most common surgical procedures requiring transfusions. However, advances in medical technology have led to a decline in reported transfusion rates over time, with rates decreasing from 21.4% in 2011 to 2.5% in 2019 for THA and from 17.6 to 0.7% for TKA during the same period (9). To investigate the potential adverse outcomes associated with perioperative allogeneic transfusion, the present study used the TriNetX global research database to evaluate postoperative risk events, including the need for retransfusion, urinary tract infection, postoperative infection, sepsis, pulmonary embolism, deep vein thrombosis (DVT), and mortality.

## 2 Materials and methods

### 2.1 Data sources

The data used in this study were collected on Oct 15, 2024 from the TriNetX global database, which provided access to electronic medical records—including diagnoses, procedures, medications, laboratory values, genomic information—from approximately 323,669 patients across 126 healthcare organizations. TriNetX is a global federated research platform that aggregates real-world clinical data from healthcare providers in more than 25 countries, enabling large-scale retrospective analyses with substantial statistical power. The database includes comprehensive diagnostic coding, treatment records, demographic information, and longitudinal follow-up data, ensuring robust capture of clinical events. All data accessed in this study were fully de-identified in compliance with the Health Insurance Portability and Accountability Act (HIPAA). Personal identifiers (e.g., names, addresses, and social security numbers) were removed or masked prior to analysis, ensuring that no individual could be directly or indirectly identified.

### 2.2 Cohort definition

**Figure 1:**
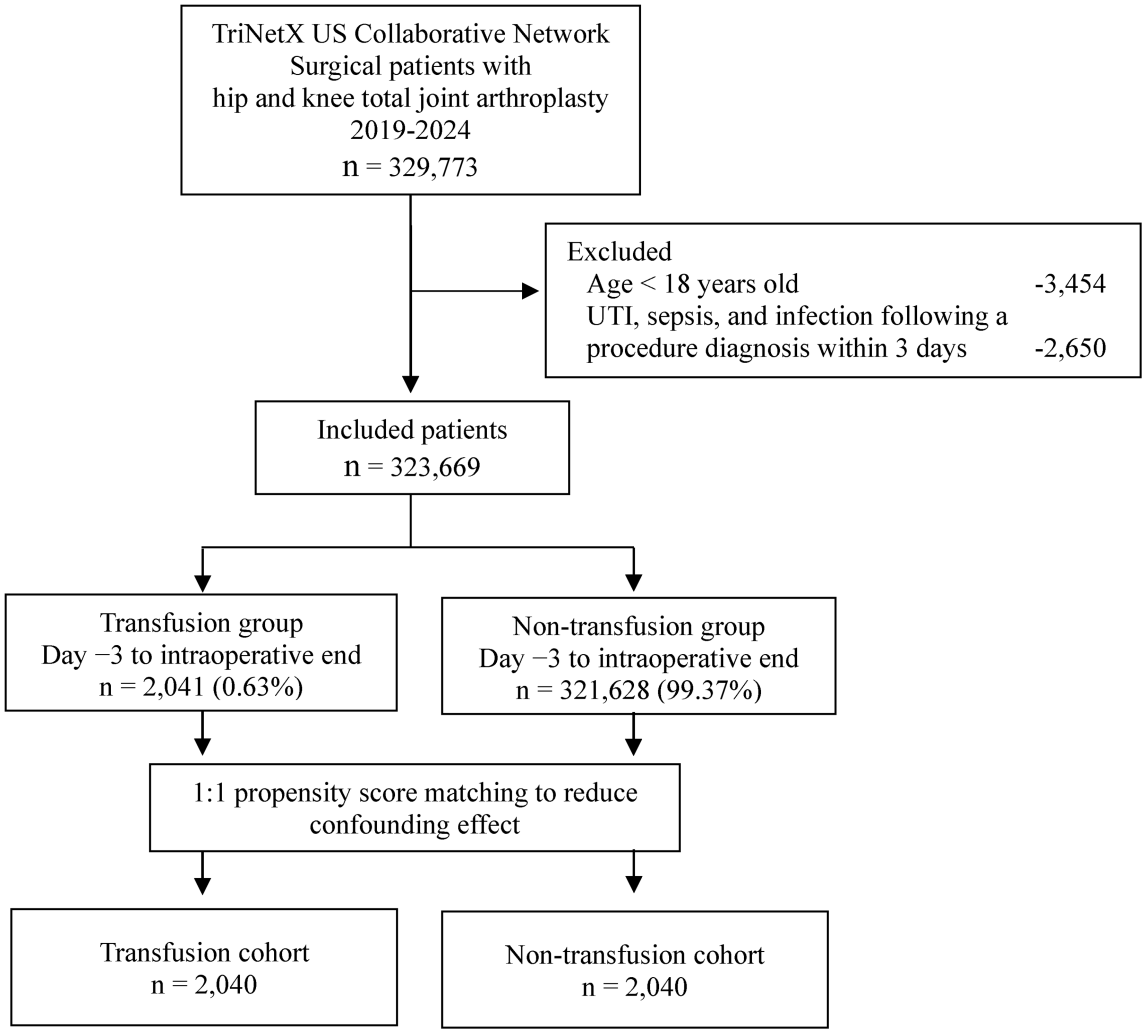
Flowchart of the patient selection and analysis process in the TriNetX database. This chart outlines the exclusion criteria applied to identify the final study cohort. The analysis included patients who underwent total hip arthroplasty (THA) and total knee arthroplasty (TKA) between 2019 and 2024. The patients were divided into transfusion and non-transfusion cohorts based on whether they received any allogenic blood transfusion from 3 days before surgery through the end of the intraoperative period. To minimize confounding effects, 1:1 PSM was applied. TKA, total knee arthroplasty; THA, total hip arthroplasty; UTI, urinary tract infection.

A retrospective analysis was conducted on 329,773 cases of THA (CPT codes: 27130) and TKA (CPT codes: 27447) performed between January 2019 and September 2024. Patients with any documented infectious diagnosis during the 3 days prior to surgery were excluded to minimize reverse causality and ensure that only postoperative infections were captured. This 72-h timeframe aligns with standard preoperative admission practices for elective joint replacement surgery (10) and is consistent with methodological approaches used in previous perioperative transfusion research (11). Patients younger than 18 years (*n* = 3,454) and cases with a documented diagnoses of urinary tract infection, sepsis, or surgical site infection within 3 days prior to surgery (*n* = 2,650) were excluded, resulting in a final study cohort of 323,669 individuals. The cohort was divided into two groups: a perioperative blood transfusion group and a non-transfusion control group. Perioperative transfusion was defined as transfusions administered from 3 day prior to surgery through the intraoperative period. Perioperative blood transfusion, as defined in this study, included allogeneic transfusion of red blood cells, platelets, or plasma administered within 3 days preoperatively or intraoperatively. To minimize the effect of confounding factors, including demographic variables, race, and comorbidities, 1:1 propensity score matching (PSM) was employed to reduce selection bias (Figure 1). Following PSM, the final matched cohort comprised 2,040 patients in the transfusion group and 2,040 in the control group.

### 2.3 Laboratory parameters

Laboratory data were collected at two time points: the most recent preoperative measurement and the first available postoperative measurement. The analyzed parameters included hemoglobin concentration, hematocrit, platelet count, international normalized ratio (INR), activated partial thromboplastin time (aPTT), and prothrombin time (PT). Additionally, body mass index (BMI) was calculated for each patient on the basis of their recorded height and weight.

### 2.4 Outcome measurement

Postoperative adverse events were identified using ICD-10 primary or secondary diagnosis codes recorded from postoperative day 1 through day 30 after the index arthroplasty procedure. Patients with documented infectious diagnoses prior to or on the day of surgery were excluded to reduce misclassification of pre-existing conditions. The primary outcome of this study was the occurrence of postoperative blood transfusion, infection, or hematological disorder after surgery. Major diagnosis was identified using the following *International Classification of Diseases*, *Tenth Revision (ICD-10)* codes: Urinary tract infection (N39.0), sepsis (A41.9), postoperative infection (T81.4), acute embolism and thrombosis of unspecified deep veins of the lower extremity (I82.40), and pulmonary embolism (I26). All patients were followed longitudinally for 30 days postoperatively to capture these outcomes.

### 2.5 Statistical analysis

All statistical analyses were performed using the TriNetX cloud-based research platform (TriNetX, LLC, 100 Cambridgepark Drive, Suite 501, Cambridge, MA 02140, United States), which utilizes validated analytic engines operating on fully de-identified electronic health records. To minimize confounding by indication, propensity score matching (PSM) was conducted using a logistic regression model including 20 predefined demographic and clinical variables. A 1:1 nearest-neighbor matching strategy with a caliper of 0.1 SD on the logit of the propensity score was applied without replacement. Covariate balance was assessed using standardized mean differences (SMDs), with SMD < 0.1 indicating adequate balance between cohorts. After matching, Cox proportional hazards regression was used to estimate hazard ratios (HRs) and 95% confidence intervals (CIs) for each postoperative outcome. All statistical tests were two-tailed, and a *p*-value < 0.05 was considered statistically significant.

## 3 Results

### 3.1 Baseline characteristics

This retrospective study analyzed data from the TriNetX online research platform to investigate the association between perioperative blood transfusion and 30-day postoperative adverse events in patients undergoing TKA and THA. Among 323,669 included patients, 2,041 (0.63%) received allogeneic blood transfusions during the perioperative period. To minimize confounding, 1:1 propensity score matching was performed to adjust for demographic variables, including age, sex, race, and relevant comorbidities. Table 1 presents the baseline characteristics of the participants. The mean age of the patients in the transfusion group was 66.5 years, with 1,221 (59.8%) being women. Prior to matching, significant differences were observed between the transfusion and control groups in terms of age, sex, and race. However, after matching, no significant differences in demographics or comorbidities were noted between the two groups.

**Table 1:** Baseline characteristics of study participants (before and after propensity score matching).

Characteristic	Before matching			After matching		
	Transfusion *n* = 2,041	Non-transfusion *n* = 321,628	SMD	Transfusion *n* = 2,040	Non-transfusion *n* = 2,040	SMD
Age (years)	66.5 ± 12.8	66.4 ± 10.0	0.007	66.5 ± 12.8	66.9 ± 12.2	0.027
Gender						
Male	803 (39.3%)	127,239 (39.6%)	0.004	803 (39.4%)	814 (39.9%)	0.011
Female	1,221 (59.8%)	175,710 (54.6%)	**0.105**	1,220 (59.8%)	1,208 (59.2%)	0.012
Race						
White	1,632 (80.0%)	244,375 (76.0%)	0.096	1,631 (80.0%)	1,683(82.5%)	0.065
Black or African American	243 (11.9%)	30,086 (9.4%)	0.083	243 (11.9%)	218 (10.7%)	0.039
Asian	62 (3.0%)	6,408 (2.0%)	0.067	62 (3.0%)	52 (2.5%)	0.030
Not Hispanic or Latino	1,710 (83.8%)	254,227 (79.0%)	**0.122**	1,710 (83.8%)	1,717 (84.2%)	0.009
Unknown ethnicity	244 (12.0%)	53,058 (16.5%)	**0.130**	244 (12.0%)	240 (11.8%)	0.006
Hispanic or Latino	87 (4.3%)	14,343 (4.5%)	0.010	86 (4.2%)	83 (4.1%)	0.007
Unknown race	61 (3.0%)	29,892 (9.3%)	**0.265**	61 (3.0%)	53 (2.6%)	0.024
Other race	29 (1.4%)	9,392 (2.9%)	**0.103**	29 (1.4%)	23 (1.1%)	0.026
Native Hawaiian or Other Pacific Islander	10 (0.5%)	758 (0.2%)	0.042	10 (0.5%)	10 (0.5%)	<0.001
American Indian or Alaska Native	10 (0.5%)	717 (0.2%)	0.045	10 (0.5%)	10 (0.5%)	<0.001
Comorbidities						
Osteoarthritis	1,511 (74.0%)	279,184 (86.8%)	**0.326**	1,511 (74.1%)	1,524 (74.7%)	0.015
Inflammatory polyarthropathy	539 (26.4%)	70,514 (21.9%)	**0.105**	539 (26.4%)	543 (26.6%)	0.004
Diabetes mellitus	639 (31.3%)	58,158 (18.1%)	**0.310**	638 (31.3%)	660 (32.4%)	0.023
Aplastic anemia and bone marrow failure	1,317 (64.5%)	51,097 (15.9%)	**1.142**	1,316 (64.5%)	1,313 (64.4%)	0.003
Coagulation defect, purpura and hemorrhage	544 (26.7%)	17,833 (5.5%)	**0.600**	543 (26.6%)	558 (27.4%)	0.017
Disorders of blood and blood-forming organs	584 (28.6%)	28,196 (8.8%)	**0.526**	583 (28.6%)	586 (28.7%)	0.003

Standardized mean differences (SMD); bold font represents a standardized difference >0.1.

### 3.2 Clinical parameters before surgery

**Figure 2:**
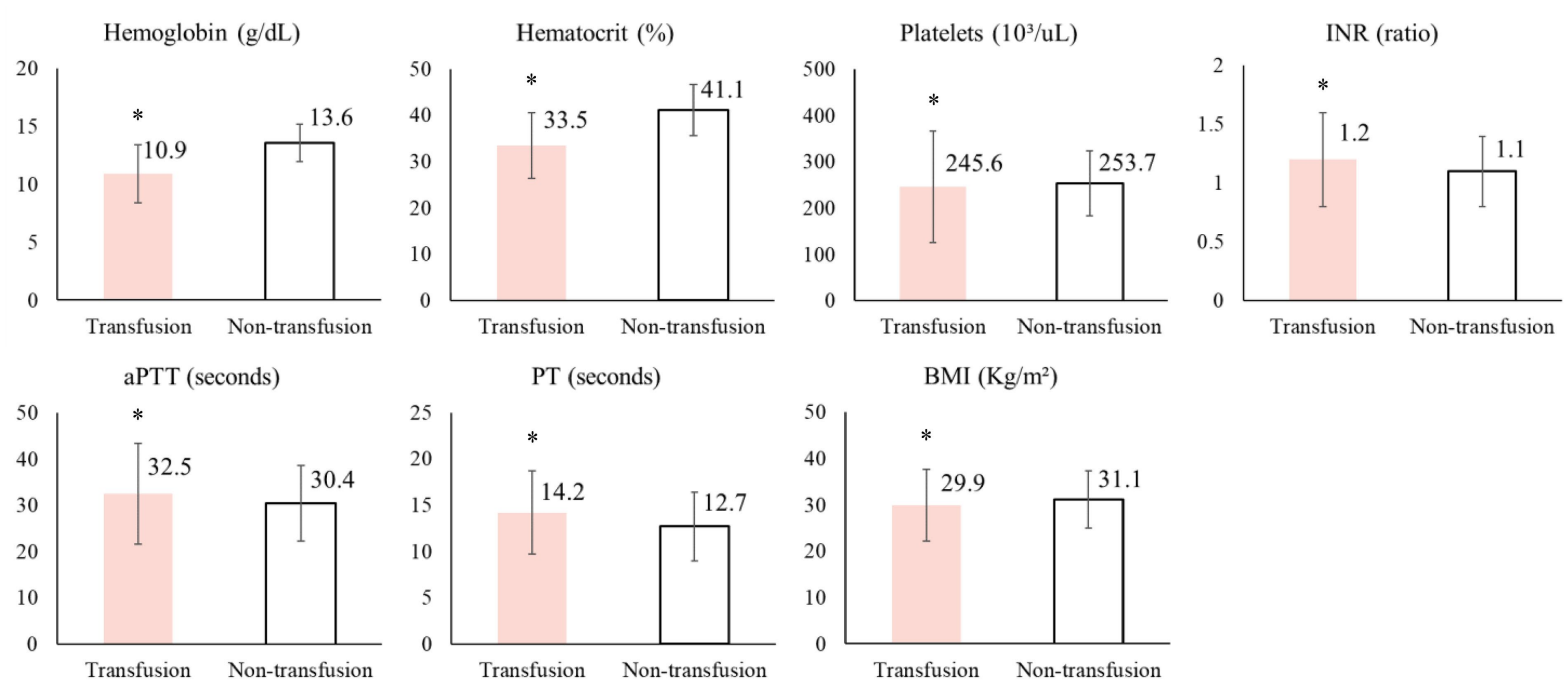
Comparison of clinical parameters among study participants. Hemoglobin concentration (g/dL), hematocrit (%), platelet count (10^3^/μL), international normalized ratio (INR), activated partial thromboplastin time (aPTT, in seconds), prothrombin time (PT, in seconds), and body mass index (BMI; kg/m^2^). Continuous variables are reported as mean ± SDs, whereas categorical variables are reported as numbers (%); Student’s *t*-test was used to compare continuous variables. *p < 0.001.

To further characterize the study population, the clinical parameters were compared between the perioperative blood transfusion group and the non-transfusion group, as presented in Figure 2. Patients in the perioperative blood transfusion group had significantly lower hemoglobin levels (10.9 ± 2.5 g/dL) and hematocrit values (33.5 ± 7.1%) compared to those in the non-transfusion group (hemoglobin: 13.6 ± 1.6 g/dL; hematocrit: 41.1 ± 5.5%, *p* < 0.001 for both). Moreover, the platelet counts were lower in the transfusion group (245.6 ± 119.9 × 10^3^/μL) than in the non-transfusion group (253.7 ± 69.7 × 10^3^/μL, *p* < 0.001), and the transfusion group exhibited significantly higher INR values (1.2 ± 0.4) than did the non-transfusion group (1.1 ± 0.3, *p* < 0.001). Regarding coagulation times, the transfusion group exhibited prolonged aPTT (32.5 ± 10.9 s vs. 30.4 ± 8.2 s, *p* < 0.001) and PT (14.2 ± 4.5 s vs. 12.7 ± 3.7 s, *p* < 0.001) relative to the non-transfusion group. Additionally, the patients in the transfusion group had a lower BMI (29.9 ± 7.8) than that of those in the non-transfusion group (31.1 ± 6.2, *p* < 0.001).

### 3.3 Adverse outcomes within 30 days

**Figure 3:**
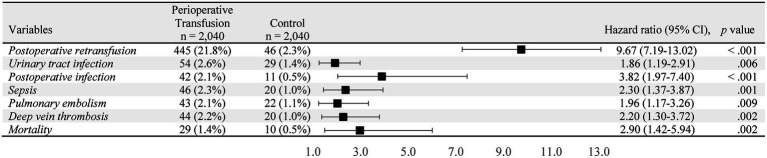
Association between perioperative blood transfusion and adverse outcomes within 30 days after total joint replacement surgery. Hazard ratios (HRs) with 95% confidence intervals (CIs) and *p*-values are presented for postoperative retransfusion, urinary tract infection, postoperative infection, sepsis, pulmonary embolism, deep vein thrombosis, and mortality.

The association between perioperative blood transfusion and adverse outcomes within 30 days after total joint replacement is presented in Figure 3. Patients who received perioperative transfusions exhibited a substantially higher risk of requiring an additional transfusion compared with those without transfusion exposure (HR = 9.67; 95% CI, 7.19–13.02; *p* < 0.001). Moreover, these patients exhibited an increased risk of infection-related complications, including urinary tract infection (HR = 1.86; 95% CI, 1.19–2.91, *p* = 0.006), postoperative infection (HR = 3.82; 95% CI, 1.97–7.40; *p* < 0.001), and sepsis (HR = 2.30; 95% CI, 1.37–3.87; *p* = 0.001). The transfusion group also had a higher incidence of thrombotic events, including a significantly increased risk of pulmonary embolism (HR = 1.96; 95% CI, 1.17–3.26; *p* = 0.009) and DVT (HR = 2.20; 95% CI, 1.30–3.72; *p* = 0.002). Additionally, perioperative transfusion was associated with a significantly increased risk of 30-day mortality compared with the non-transfusion group (HR = 2.90; 95% CI, 1.42–5.94; *p* = 0.002).

## 4 Discussion

This study investigated the association between perioperative blood transfusion and the risk of adverse outcomes in patients undergoing THA or TKA. A retrospective cohort analysis was conducted using the TriNetX research database, which encompasses data from over 130 million patients worldwide (Figure 1). The findings indicated a relatively low perioperative transfusion rate among patients undergoing total joint replacement. Analysis of 30-day postoperative outcomes revealed that perioperative blood transfusion was associated with an increased likelihood of retransfusion as well as a higher risk of complications, including urinary tract infections, surgical site infections, sepsis, pulmonary embolism, venous thrombosis, and mortality (Figure 3).

Total joint arthroplasty is commonly associated with substantial blood loss, which may lead to a decline in hemoglobin levels and increase the likelihood of allogeneic blood transfusion. Although the incidence of substantial blood loss during these procedures has markedly declined since 2000 (12, 13), blood loss remains a critical concern in perioperative management. In the present cohort of 323,669 patients undergoing total joint replacement, only 2,041 required transfusion, resulting in a perioperative transfusion rate of 0.63%, which is considerably lower than rates reported in earlier studies (14). This reduction may be attributed to advances in medical technology and perioperative care. Key innovations include minimally invasive surgical techniques that reduce tissue trauma and blood loss (15), the widespread use of tranexamic acid as a hemostatic agent (14, 16, 17), optimized intraoperative blood pressure management, and preoperative interventions such as iron supplementation or erythropoietin administration to improve hemoglobin levels. In addition, the implementation of comprehensive patient blood management (PBM) programs has played a pivotal role in minimizing unnecessary transfusions (18). Nevertheless, despite perioperative allogeneic blood transfusions remains necessary in a subset of patients, underscoring the importance of evaluating its downstream clinical consequences.

Comparison of laboratory data between patients who received perioperative transfusion and those who not showed significantly lower preoperative hemoglobin and hematocrit levels in the transfusion group, indicating that these patients were more likely to require transfusion. Declining preoperative hemoglobin levels are strongly associated with increased transfusion risk, highlighting the importance of identifying and correcting anemia prior to surgery (19). Previous studies have demonstrated that preoperative anemia is an independent risk factor for allogeneic blood transfusion (20), postoperative infections (21) and mortality (22). With respect to coagulation-related parameters, patients who received transfusion exhibited prolonged preoperative PT, aPTT, and INR, emphasizing the role of transfusion in maintaining perioperative hemostatic stability. Prior research has also shown that female sex and lower BMI are associated with an increased risk of postoperative transfusion, particularly following hip surgery (23).

The present study further demonstrated that perioperative transfusion was associated with a substantially higher probability of postoperative retransfusion within 30 days (Figure 3). This finding is consistent with previous evidence indicating that preoperative anemia and greater underlying surgical severity contribute to increased transfusion demand (12). Moreover, an ACS-NSQIP-based study reported that preoperative transfusion administered within 72 h before revision TKA was independently associated with increased postoperative complications, including myocardial infarction and postoperative transfusion, even after adjustment for comorbidities (11). Perioperative transfusion may therefore serve as a surrogate marker of more severe clinical status. Older adults with undiagnosed anemia identified through preadmission testing, as well as critically ill patients experiencing major fractures or intraoperative hemorrhage, often require transfusion. In addition, cardiovascular and renal comorbidities may exacerbate anemia and further increase transfusion demand. However, preoperative transfusions may not fully restore circulating blood volume or hemoglobin levels, resulting in additional postoperative transfusions events. The elevated postoperative infection risk observed among transfused patients may be partially explained by transfusion-related immunomodulation (TRIM), whereby allogeneic leukocytes and bioactive mediators impair innate immune responses, including antigen presentation and natural killer cell activity, thereby increasing susceptibility to pathogens. These mechanisms are consistent with meta-analyses in orthopedic surgery demonstrating a significantly increased risk of postoperative infection following transfusion (OR 2.99; 95% CI, 1.95–4.59) (24).

The present study found that patients who received perioperative allogeneic blood transfusions had a higher risk of postoperative infections compared with those who did not receive transfusion. This association aligns with prior evidence linking transfusion exposure to increased susceptibility to urinary tract infections, surgical site infections, and sepsis (8, 25). Postoperative infections after joint replacement are clinically significant (26), as periprosthetic joint infections are difficult to manage and are associated with substantial morbidity, impaired quality of life, and increased mortality (27). In addition, the treatment of prosthetic joint infections imposes a considerable economic burden on healthcare systems (28, 29).

Our analysis also demonstrated a significant association between perioperative allogeneic blood transfusion and postoperative deep vein thrombosis in patients undergoing THA and TKA (HR = 2.20; 95% CI, 1.30–3.72; *p* = 0.002). Similarly, Jiang et al. reported a fourfold increased risk of postoperative DVT among transfused patients (30). This association may be related to transfusion-induced alterations in coagulation pathways and increased blood viscosity, both of which contribute to an elevate risk of venous thromboembolism, including pulmonary embolism. These findings are consistent with previous studies highlighting the hemostatic and immunological effects of transfusion on postoperative outcomes.

Overall, these findings underscore the necessity of implementing stringent perioperative transfusion practices. Comprehensive PBM strategies are essential for minimizing blood loss, optimizing preoperative hemoglobin levels, and applying evidence-based transfusion thresholds. By reducing the reliance on allogeneic blood transfusion, such approaches may help mitigate transfusion-related complications and improve postoperative outcomes in patients undergoing total joint arthroplasty.

## 5 Summary

Perioperative allogeneic blood transfusion was significantly associated with increased risks of postoperative transfusion, infections complications, thromboembolic events, and mortality among patients undergoing THA or TKA.

## 6 Limitations

Despite the advantages of leveraging a large-scale international research network, this study has several limitations related to the use of the TriNetX platform. First, the representativeness of the dataset may be limited, as TriNetX aggregates data primarily from participating health-care institutions and may not fully capture the demographic and clinical characteristics of broader populations. Second, missing or incomplete data may have introduced information bias and affected the accuracy of outcome assessment. Third, although smoking and alcohol use data were available in the database, these lifestyle factors were not included because they are less likely to influence transfusion decisions within the short perioperative exposure window; however, their omission may still contribute to residual confounding, along with other unmeasured variable such as socioeconomic status, insurance coverage, and detailed clinical information. Although extensive propensity score matching was performed to reduce confounding, unmeasured confounders may still have influenced the observed associations. Fourth, analyses were restricted to the built-in statistical tools available within the TriNetX platform, limiting the application of more advanced or customized modeling strategies. In addition, residual immortal time bias may persist, as patients requiring earlier perioperative transfusion could differ in baseline severity from those transfused closer to surgery, even though the index date was anchored to the day of surgery to mitigate this concern. These limitations should be considered when interpreting the finding. Future studies integrating more granular clinical datasets and advanced analytic methods are warranted to validate and extend our findings.

## Data Availability

The datasets presented in this study can be found in online repositories. The names of the repository/repositories and accession number(s) can be found in the article/supplementary material.
